# Feigenbaum Graphs: A Complex Network Perspective of Chaos

**DOI:** 10.1371/journal.pone.0022411

**Published:** 2011-09-07

**Authors:** Bartolo Luque, Lucas Lacasa, Fernando J. Ballesteros, Alberto Robledo

**Affiliations:** 1 Department of Matemática Aplicada y Estadística, ETSI Aeronáuticos, Universidad Politécnica de Madrid, Madrid, Spain; 2 Observatori Astronòmic, Universitat de València, València, Spain; 3 Department of Matemáticas, Universidad Carlos III de Madrid, Madrid, Spain; 4 Instituto de Física, Universidad Nacional Autónoma de México, México, D.F., Mexico; University of Zaragoza, Spain

## Abstract

The recently formulated theory of horizontal visibility graphs transforms time series into graphs and allows the possibility of studying dynamical systems through the characterization of their associated networks. This method leads to a natural graph-theoretical description of nonlinear systems with qualities in the spirit of symbolic dynamics. We support our claim via the case study of the period-doubling and band-splitting attractor cascades that characterize unimodal maps. We provide a universal analytical description of this classic scenario in terms of the horizontal visibility graphs associated with the dynamics within the attractors, that we call Feigenbaum graphs, independent of map nonlinearity or other particulars. We derive exact results for their degree distribution and related quantities, recast them in the context of the renormalization group and find that its fixed points coincide with those of network entropy optimization. Furthermore, we show that the network entropy mimics the Lyapunov exponent of the map independently of its sign, hinting at a Pesin-like relation equally valid out of chaos.

## Introduction

We expose a remarkable relationship between nonlinear dynamical systems and complex networks by means of the horizontal visibility (HV) algorithm [1]–[3] that transforms time series into graphs. In low-dimensional dissipative systems chaotic motion develops out of regular motion in a small number of ways or routes, and amongst which the period-doubling bifurcation cascade or Feigenbaum scenario is perhaps the better known and most famous mechanism [4], [5]. This route to chaos appears an infinite number of times amongst the family of attractors spawned by unimodal maps within the so-called periodic windows that interrupt stretches of chaotic attractors. In the opposite direction, a route out of chaos accompanies each period-doubling cascade by a chaotic band-splitting cascade, and their shared bifurcation accumulation points form transitions between order and chaos that are known to possess universal properties [4]–[6]. Low-dimensional maps have been extensively studied from a purely theoretical perspective, but systems with many degrees of freedom used to study diverse problems in physics, biology, chemistry, engineering, and social science, are known to display low-dimensional dynamics [7].

The horizontal visibility (HV) algorithm converts the information stored in a time series into a network, setting the nature of the dynamical system into a different context that requires complex network tools [8]–[12] to extract its properties. This approach belongs to an emerging corpus of methods that map series to networks (see for instance [2], [13]–[17] or a recent review [18]). Relevant information can be obtained through the family of visibility methods, including the characterization of fractal behavior [19] or the discrimination between random and chaotic series [1], [20], and it finds increasing applications in separate fields, from geophysics [21], to finance [22] or physiology [23]. Here we offer a distinct view of the Feigenbaum scenario through the specific HV formalism, and provide a complete set of graphs, which we call Feigenbaum graphs, that encode the dynamics of all stationary trajectories of unimodal maps. We first characterize their topology via the order-of-visit and self-affinity properties of the maps. Additionally, a matching renormalization group (RG) procedure leads, via its flows, to or from network fixed-points to a comprehensive view of the entire family of attractors. Furthermore, the optimization of the entropy obtained from the degree distribution coincides with the RG fixed points and reproduces the essential features of the map's Lyapunov exponent independently of its sign. A general observation is that the HV algorithm extracts only universal elements of the dynamics, free of the peculiarities of the individual unimodal map, but also of universality classes characterized by the degree of nonlinearity. Therefore all the results presented in this work, while referring to the specific Logistic map for illustrative reasons apply to any unimodal map.

**Model: Feigenbaum graphs**

**Figure 1:**
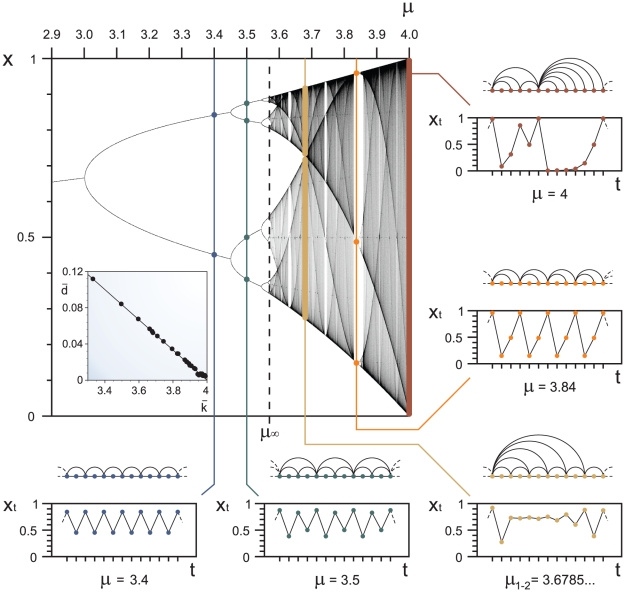
Feigenbaum graphs from the Logistic map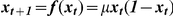. The main figure portrays the family of attractors of the Logistic map and indicates a transition from periodic to chaotic behavior at 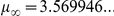 through period-doubling bifurcations. For 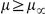 the figure shows merging of chaotic-band attractors where aperiodic behavior appears interrupted by windows that, when entered from their left-hand side, display periodic motion of period 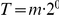 with 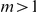 (for 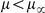, 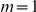) that subsequently develops into 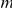 period-doubling cascades with new accumulation points 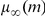. Each accumulation point 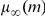 is in turn the limit of a chaotic-band reverse bifurcation cascade with 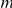 initial chaotic bands, reminiscent of the self-affine structure of the entire diagram. All unimodal maps exhibit a period-doubling route to chaos with universal asymptotic scaling ratios between successive bifurcations that depend only on the order of the nonlinearity of the map [30], the Logistic map belongs to the quadratic case. Adjoining the main figure, we show time series and their associated Feigenbaum graphs according to the HV mapping criterion for several values of 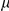 where the map evidences both regular and chaotic behavior (see the text). *Inset:* Numerical values of the mean normalized distance 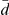 as a function of mean degree 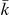 of the Feigenbaum graphs for 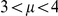 (associated to time series of 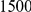 data after a transient and a step 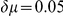), in good agreement with the theoretical linear relation (see the text).

The HV graph [1] associated with a given time series 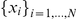 of 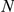 real data is constructed as follows: First, a node 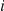 is assigned to each datum 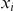, and then two nodes 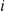 and 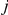 are connected if the corresponding data fulfill the criterion 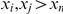 for all 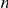 such that 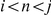. Let us now focus on the Logistic map [4] defined by the quadratic difference equation 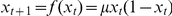 where 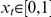 and the control parameter 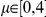. According to the HV algorithm, a time series generated by the Logistic map for a specific value of 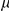 (after an initial transient of approach to the attractor) is converted into a Feigenbaum graph (see figure 1). Notice that this is a well-defined subclass of HV graphs where consecutive nodes of degree 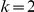, that is, consecutive data with the same value, do not appear, what is actually the case for series extracted from maps (besides the trivial case of a constant series). While for a period 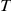 there are in principle several possible periodic orbits, and therefore the set of associated Feigenbaum graphs is degenerate, it can be proved that the mean degree 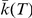 and normalized mean distance 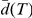 of all these Feigenbaum graphs fulfill 
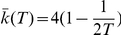
 and 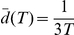 respectively, yielding a linear relation 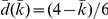 that is corroborated in the inset of figure 1. Observe that aperiodic series (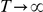) reach the upper bound mean degree 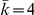.

## Results

**Figure 2:**
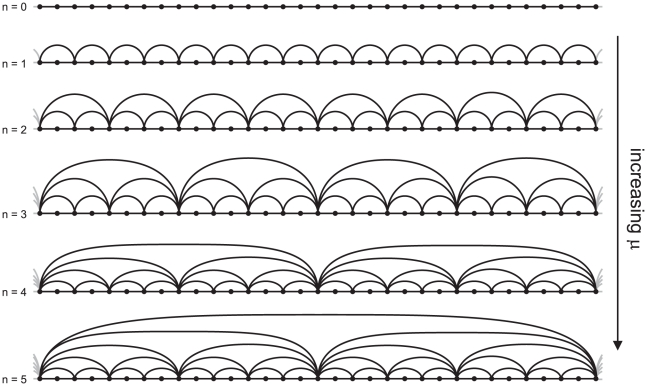
Periodic Feigenbaum graphs for 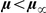. The sequence of graphs associated to periodic attractors with increasing period 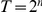 undergoing a period-doubling cascade. The pattern that occurs for increasing values of the period is related to the universal ordering with which an orbit visits the points of the attractor. Observe that the hierarchical self-similarity of these graphs requires that the graph for 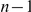 is a subgraph of that for 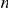.

A deep-seated feature of the period-doubling cascade is that the order in which the positions of a periodic attractor are visited is universal [24], the same for all unimodal maps. This ordering turns out to be a decisive property in the derivation of the structure of the Feigenbaum graphs. See figure 2 where we plot the graphs for a family of attractors of increasing period 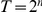, that is, for increasing values of 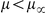. This basic pattern also leads to the expression for their associated degree distributions,
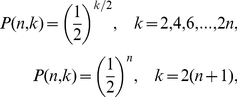
(1) and zero for 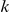 odd or 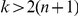. At the accumulation point 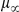 the period diverges (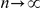) and the distribution is exponential for all even values of the degree, 
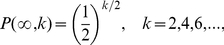
(2)and zero for 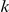 odd. Observe that these relations are independent of the order of the map's nonlinearity: the HV algorithm sifts out every detail of the dynamics except for the basic storyline.

**Figure 3:**
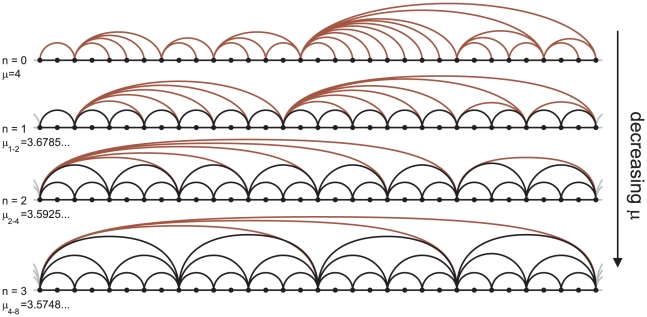
Aperiodic Feigenbaum graphs for 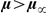. A sequence of graphs associated with chaotic series after 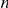 chaotic-band reverse bifurcations, starting at 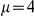 for 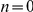, when the attractor extends along a single band and the degree distribution does not present any regularity (red links). For 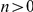 the phase space is partitioned in 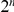 disconnected chaotic bands and the 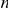-th self-affine image of 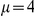 is the 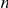-th Misiurewicz point 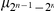. In all cases, the orbit visits each chaotic band in the same order as in the periodic region 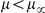. This order of visits induces an ordered structure in the graphs (black links) analogous to that found for the period-doubling cascade.

We turn next to the period-doubling bifurcation cascade of chaotic bands that takes place as 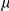 decreases from 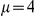 towards 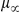. For the largest value of the control parameter, at 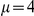, the attractor is fully chaotic and occupies the entire interval 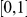 (see figure 1). This is the first chaotic band 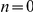 at its maximum amplitude. As 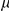 decreases in value within 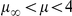 band-narrowing and successive band-splittings [4]–[6], [24] occur. In general, after 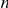 reverse bifurcations the phase space is partitioned in 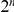 disconnected chaotic bands, which are self-affine copies of the first chaotic band [25]. The values of 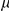 at which the bands split are called Misiurewicz points [24], and their location converges to the accumulation point 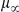 for 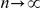. Significantly, while in the chaotic zone orbits are aperiodic, for reasons of continuity they visit each of the 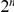 chaotic bands in the same order as positions are visited in the attractors of period 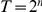 [24]. In figure 3 we have plotted the Feigenbaum graphs generated through chaotic time series at different values of 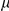 that correspond to an increasing number of reverse bifurcations. Since chaotic bands do not overlap, one can derive the following degree distribution for a Feigenbaum graph after 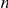 chaotic-band reverse bifurcations by using only the universal order of visits
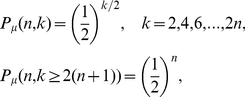
(3) and zero for 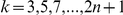. We note that this time the degree distribution retains some dependence on the specific value of 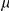, concretely, for those nodes with degree 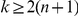, all of which belong to the top chaotic band (labelled with red links in figure 3). The HV algorithm filters out chaotic motion within all bands except for that taking place in the top band whose contribution decreases as 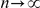 and appears coarse-grained in the cumulative distribution 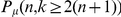. As would be expected, at the accumulation point 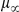 we recover the exponential degree distribution (equation 2), *i.e.* 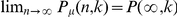.

Before proceeding to interpret these findings via the consideration of renormalization group (RG) arguments, we recall that the Feigenbaum tree shows a rich self-affine structure: for 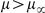 periodic windows of initial period 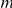 undergo successive period-doubling bifurcations with new accumulation points 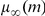 that appear interwoven with chaotic attractors. These cascades are self-affine copies of the fundamental one. The process of reverse bifurcations also evidences this self-affine structure, such that each accumulation point is the limit of a chaotic-band reverse bifurcation cascade. Accordingly, we label 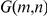 the Feigenbaum graph associated with a periodic series of period 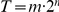, that is, a graph obtained from an attractor within window of initial period 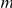 after 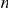 period-doubling bifurcations. In the same fashion, 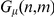 is associated with a chaotic attractor composed by 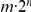 bands (that is, after 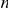 chaotic band reverse bifurcations of 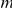 initial chaotic bands). Therefore, graphs depicted in figures 2 and 3 correspond to 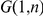 and 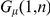 respectively and for the first accumulation point we have 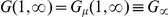. Similarly, in each accumulation point 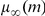 we have 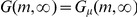.

**Figure 4:**
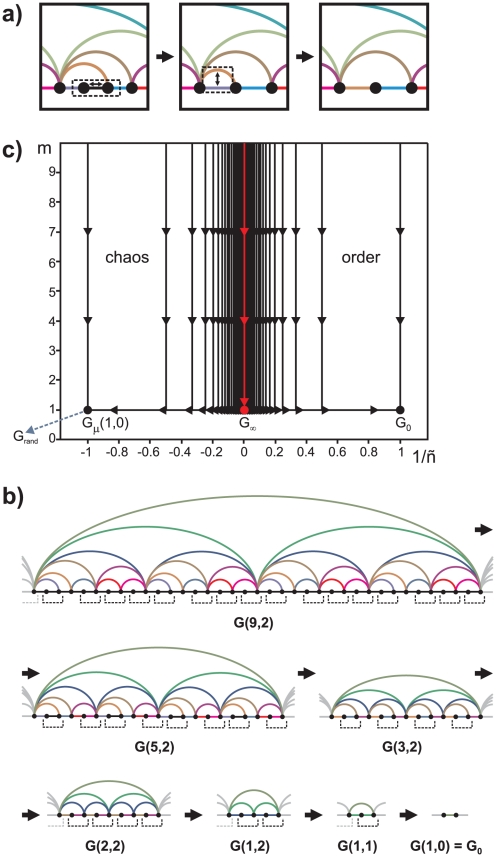
Renormalization process and network RG flow structure. (a) Illustration of the renormalization process 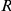: a node with degree 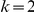 is coarse-grained with one of its neighbors (indistinctively) into a block node that inherits the links of both nodes. This process coarse-grains every node with degree 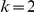 present at each renormalization step. (b) Example of an iterated renormalization process in a sample Feigenbaum graph at a periodic window with initial period 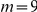 after 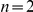 period-doubling bifurcations (an orbit of period 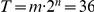). (c) RG flow diagram, where 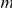 identifies the periodic window that is initiated with period 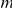 and *ñ* designates the order of the bifurcation, *ñ* 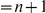 for period-doubling bifurcations and *ñ* 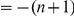 for reverse bifurcations. 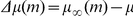 denotes the reduced control parameter of the map, and 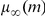 is the location of the accumulation point of the bifurcation cascades within that window. Feigenbaum graphs associated with periodic series (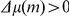, *ñ* 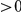) converge to 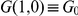 under the RG, whereas those associated with aperiodic ones (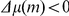, *ñ* 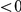) converge to 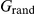. The accumulation point 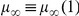 corresponds to the unstable (nontrivial) fixed point 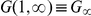 of the RG flow, which is nonetheless approached through the critical manifold of graphs 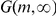 at the accumulation points 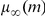. In summary, the nontrivial fixed point of the RG flow is only reached via the family of the accumulation points, otherwise the flow converges to trivial fixed points for periodic or chaotic regions.

In order to recast previous findings in the context of the renormalization group, let us define an RG operation 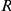 on a graph as the coarse-graining of every couple of adjacent nodes where one of them has degree 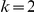 into a block node that inherits the links of the previous two nodes (see figure 4.a). This is a real-space RG transformation on the Feigenbaum graph [26], dissimilar from recently suggested box-covering complex network renormalization schemes [27], [28], [29]. This scheme turns out to be equivalent for 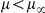 to the construction of an HV graph from the composed map 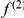 instead of the original 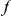, in correspondence to the original Feigenbaum renormalization procedure [30], [6]. We first note that 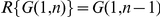, thus, an iteration of this process yields an RG flow that converges to the (1st) trivial fixed point 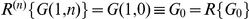. This is the stable fixed point of the RG flow 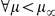. We note that there is only one relevant variable in our RG scheme, represented by the reduced control parameter 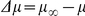, hence, to identify a nontrivial fixed point we set 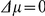 or equivalently 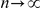, where the structure of the Feigenbaum graph turns to be completely self-similar under 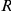. Therefore we conclude that 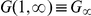 is the nontrivial fixed point of the RG flow, 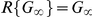. In connection with this, let 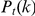 be the degree distribution of a generic Feigenbaum graph 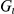 in the period-doubling cascade after 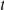 iterations of 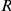, and point out that the RG operation, 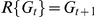, implies a recurrence relation 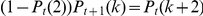, whose fixed point coincides with the degree distribution found in equation 2. This confirms that the nontrivial fixed point of the flow is indeed 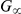.

Next, under the same RG transformation, the self-affine structure of the family of attractors yields 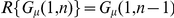, generating a RG flow that converges to the Feigenbaum graph associated to the 1st chaotic band, 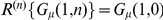. Repeated application of 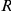 breaks temporal correlations in the series, and the RG flow leads to a 2nd trivial fixed point 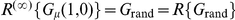, where 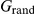 is the HV graph generated by a purely uncorrelated random process. This graph has a universal degree distribution 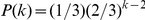, independent of the random process underlying probability density (see [1], [20]).

Finally, let us consider the RG flow inside a given periodic window of initial period 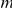. As the renormalization process addresses nodes with degree 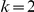, the initial applications of 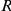 only change the core structure of the graph associated with the specific value 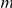 (see figure 4.b for an illustrative example). The RG flow will therefore converge to the 1st trivial fixed point via the initial path 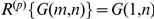, with 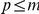, whereas it converges to the 2nd trivial fixed point for 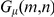 via 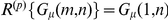. In the limit of 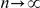 the RG flow proceeds towards the nontrivial fixed point via the path 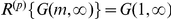. Incidentally, extending the definition of the reduced control parameter to 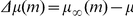, the family of accumulation points is found at 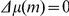. A complete schematic representation of the RG flows can be seen in figure 4.c.

Interestingly, and at odds with standard RG applications to (asymptotically) scale-invariant systems, we find that invariance at 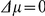 is associated in this instance to an exponential (rather than power-law) function of the observables, concretely, that for the degree distribution. The reason is straightforward: 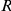 is not a conformal transformation (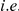 a scale operation) as in the typical RG, but rather, a translation procedure. The associated invariant functions are therefore non homogeneous (with the property 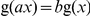), but exponential (with the property 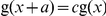).

Finally, we derive, via optimization of an entropic functional for the Feigenbaum graphs, all the RG flow directions and fixed points directly from the information contained in the degree distribution. Amongst the graph theoretical entropies that have been proposed we employ here the Shannon entropy of the degree distribution 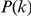, that is 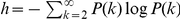. By making use of the Maximum Entropy formalism, it is easy to prove that the degree distribution 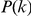 that maximizes 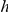 is exactly 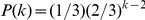, which corresponds to the distribution for the 2nd trivial fixed point of the RG flow 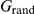. Alternatively, with the incorporation of the additional constraint that allows only even values for the degree (the topological restriction for Feigenbaum graphs 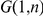), entropy maximization yields a degree distribution that coincides with equation 2, which corresponds to the nontrivial fixed point of the RG flow 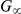. Lastly, the degree distribution that minimizes 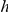 trivially corresponds to 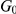, *i.e.* the 1st trivial fixed point of the RG flow. Remarkably, these results indicate that the fixed-point structure of the RG flow are obtained via optimization of the entropy for the entire family of networks, supporting a suggested connection between RG theory and the principle of Maximum Entropy [31].

**Figure 5:**
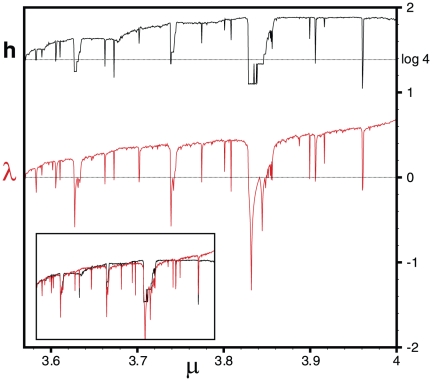
Horizontal visibility network entropy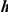 and Lyapunov exponent 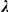 for the Logistic map. We plot the numerical values of 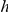 and 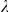 for 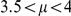 (the numerical step is 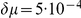 and in each case the processed time series have a size of 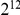 data). The inset reproduces the same data but with a rescaled entropy 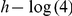. The surprisingly good match between both quantities is reminiscent of the Pesin identity (see text). Unexpectedly, the Lyapunov exponent within the periodic windows (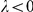 inside the chaotic region) is also well captured by 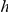.

The network entropy 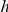 can be calculated exactly for 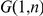 (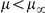 or 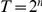), yielding 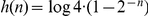. Because increments of entropy are only due to the occurrence of bifurcations 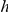 increases with 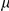 in a step-wise way, and reaches asymptotically the value 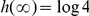 at the accumulation point 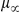. For Feigenbaum graphs 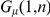 (in the chaotic region), in general 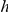 cannot be derived exactly since the precise shape of 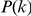 is unknown (albeit the asymptotic shape is also exponential [20]). Yet, the main feature of 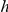 can be determined along the chaotic-band splitting process, as each reverse bifurcation generates two self-affine copies of each chaotic band. Accordingly, the decrease of entropy associated with this reverse bifurcation process can be described as 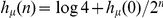, where the entropy 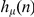 after 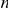 reverse bifurcations can be described in terms of the entropy associated with the first chaotic band 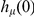. In figure 1 we observe how the chaotic-band reverse bifurcation process takes place in the chaotic region from right to left, and therefore leads in this case to a decrease of entropy with an asymptotic value of 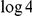 for 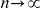 at the accumulation point. These results suggest that the graph entropy behaves qualitatively as the map's Lyapunov exponent 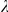, with the peculiarity of having a shift of 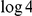, as confirmed in figure 5. This unexpected qualitative agreement is reasonable in the chaotic region in view of the Pesin theorem [5], that relates the positive Lyapunov exponents of a map with its Kolmogorov-Sinai entropy (akin to a topological entropy) that for unimodal maps reads 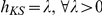, since 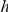 can be understood as a proxy for 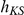. Unexpectedly, this qualitative agreement seems also valid in the periodic windows (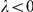), since the graph entropy is positive and varies with the value of the associated (negative) Lyapunov exponent even though 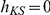, hinting at a Pesin-like relation valid also out of chaos which deserves further investigation. The agreement between both quantities lead us to conclude that the Feigenbaum graphs capture not only the period-doubling route to chaos in a universal way, but also inherits the main feature of chaos, *i.e.* sensitivity to initial conditions.

## Discussion

In summary, we have shown that the horizontal visibility theory combines power with straightforwardness as a tool for the analytical study of nonlinear dynamics. As an illustration we have established how the families of periodic and chaotic attractor bifurcation cascades of unimodal maps transform into families of networks with scale-invariant limiting forms, whose characterization can be deduced from two basic and universal properties of unimodal maps: ordering of consecutive positions in the attractors and self-affinity. Further, we have demonstrated that these networks and their associated degree distributions comply with renormalization group and maximum entropy principles, filtering out irrelevant variables and finding fixed-point networks which are independent of the map's nonlinearity. The entire Feigenbaum scenario is therefore fully described. The potential of the theory for revealing new information is indicated by the ability of the network entropy to emulate the Lyapunov exponent for both periodic and chaotic attractors. Extensions of this approach to other complex behavior, such as dynamical complexity associated to vanishing Lyapunov exponents, intermittency, quasiperiodic routes to chaos, etc., are still open questions. Finally, observe that in symbolic dynamics [32] one usually defines a phase-space partition (Markov partition) in order to create a symbolic representation of the dynamics. This partition tiles phase space in a non-overlapping manner: every value of the series has a univocally associated symbol. While a Feigenbaum graph also symbolizes the series data (incidentally, without the need of defining an ad hoc partition), each series datum is not associated univocally to a symbol (the degree of the node): this symbol is a function, in principle, of the complete series, and incorporates global information. Furthermore, note that besides the symbolization that converts a time series into a series of node degrees, the Feigenbaum graphs also store the connectivity pattern amongst nodes -i.e. the topological structure of the graph. On this respect, the possible connections of HV theory with kneading theory [33] and symbolic dynamics [32] are of special interest.
